# Noble Humbug? Hard and soft laws on clinical placebo use

**DOI:** 10.3389/fpsyg.2025.1520664

**Published:** 2025-03-12

**Authors:** Mélina Richard, Manuela Ganz, Lena Dominique Hornstein, Bohdan Stehlik, Mélanie Levy, Charlotte R. Blease, Marco Annoni, Bernice S. Elger, Jens Gaab

**Affiliations:** ^1^Institute of Biomedical Ethics, University of Basel, Basel, Switzerland; ^2^Department of Biomedical Engineering, University of Basel, Basel, Switzerland; ^3^Division of Clinical Psychology and Psychotherapy, Faculty of Psychology, University of Basel, Basel, Switzerland; ^4^Health Law Institute, Faculty of Law, University of Neuchâtel, Neuchâtel, Switzerland; ^5^Participatory eHealth and Health Data Research Group, Uppsala University, Uppsala, Sweden; ^6^Centre for Research Ethics and Integrity (CID-Ethics), National Research Council (CNR), Rome, Italy

**Keywords:** placebo, clinical placebo use, legal frameworks, clinical regulations, hard laws and soft laws, informed consent, open-label placebos (OLPs), patient rights and autonomy

## Abstract

Despite the widespread historical and contemporary use of placebos in medicine, legal regulations addressing their administration remain limited in many countries. This paper examines the legal landscape of clinical placebo use, focusing on key jurisdictions such as France, Germany, Switzerland, the United Kingdom, and the United States. Given the ethical and legal complexities surrounding placebo use, a critical assessment of existing regulatory frameworks is essential. This study employs a multidisciplinary approach, analyzing both binding laws (“hard laws”) and non-binding principles (“soft laws”) related to placebo administration. Data were collected from legal statutes, health institution guidelines, and professional medical codes to map the regulatory environment governing placebos in different legal systems. The results indicate significant variations in how placebos are addressed legally. For instance in Germany and the UK, no specific laws regulate placebo use, but statutes on informed consent implicitly cover their administration. In the United States, the American Medical Association provides ethical guidelines permitting placebo use under strict conditions emphasizing patient welfare and transparency. Across all examined jurisdictions, unauthorized placebo use may lead to legal consequences such as medical fraud allegations or violations of patient rights. These findings highlight the need for explicit regulatory guidelines to ensure that placebo use adheres to ethical and legal standards. The growing acceptance of open-label placebos (OLPs), which demonstrate efficacy without deception, presents a potential avenue for aligning legal frameworks with evolving medical practices. Future regulatory developments should address the ethical and legal challenges associated with placebos, ensuring patient autonomy and informed consent remain central to their use in clinical practice.

## 1 Introduction

A placebo is any treatment component—be it a pill, behavior, or setting—that elicits therapeutic effects through patient expectations and the context of care rather than active ingredients (Gaab et al., 2018, 2019). The use of placebos in treating ailments has a long history, deeply embedded in the fabric of medical practice, dating back to prehistoric times, and spanning various cultures around the world (Shapiro and Shapiro, 2000). This tradition underscores the placebo's role in medicine, not merely as a contemporary phenomenon but as a fundamental aspect of healing practices throughout human history. In modern clinical settings, placebos continue to play a pivotal role, with a significant prevalence of up to 95% of use among medical doctors (Linde et al., 2018) and 100% among nurses (Fässler et al., 2010). These practices include not only the application of pure placebos, which contain no active ingredients, but also so-called impure placebos, where otherwise active substances are used in a non-specific manner (Howick et al., 2013). Additionally, placebos play a crucial role in clinical trials as a control substance and in research aimed at understanding the placebo effect itself. However, this paper will focus exclusively on the clinical applications of placebo use.

Such widespread use, despite controversies surrounding the ethical implications of deceiving patients, highlights the complex interplay between clinical efficacy and moral considerations (Miller and Colloca, 2009; Wu and Stoessl, 2024). The ethical debate surrounding placebo use centers on the balance between its potential therapeutic benefits and the violation of patient autonomy, a principle deeply rooted in medical ethics (Annoni, 2018). It has been argued that there is no reason to use the placebo outside of clinical trials as there is little evidence that placebos have clinical effects (Hróbjartsson and Gøtzsche, 2001). Critics argue that deceptive placebos, by withholding information about the nature of the treatment, undermine the trust integral to the patient-physician relationship and conflict with the ethical requirement for informed consent (Bernstein et al., 2020). Yet, some practitioners and scholars advocate for the judicious use of placebos, suggesting that, under certain conditions, the therapeutic value of placebos can justify or even necessitate their use (Howick, 2023), whereas some argue that this should happen only if there is a partnership between physician and patient (Annoni and Miller, 2016; Fässler et al., 2009).

The legal and regulatory landscape regarding the use of placebo in clinical practice remains largely undefined, with few specific regulations addressing the administration of placebos within medical treatment (Aebi-Müller, 2022; Evers et al., 2018). The ongoing debate and the varied practices across different healthcare settings underscore the need for more comprehensive research and discussion on the ethical, clinical, and legal aspects of placebo use. Studies reveal a wide range of attitudes among practitioners regarding placebos, from their therapeutic efficacy to concerns over legal and ethical guidelines (Bishop et al., 2014; Fent et al., 2011). This diversity in views and practices points to the need for clearer regulations and guidelines to navigate the complex ethical landscape surrounding placebo use, ensuring that the potential benefits of placebos are harnessed in a manner that respects patient autonomy and adheres to ethical principles. Moreover, the advent of open-label placebo research challenges the notion that deception is necessary for placebos to exert their effects, offering a promising avenue for reconciling ethical concerns with the therapeutic potential of placebos (Blease, 2019; Kaptchuk et al., 2010). This emerging body of research suggests that placebos might be effective even when patients are aware of their inert nature (Buergler et al., 2023); while some have challenged these researches, they may offer new possibilities for integrating placebos into clinical practice in a more ethically and legally sound manner (Spille et al., 2023; Kaptchuk and Miller, 2018; Blease et al., 2023; Jones et al., 2023). As medicine continues to evolve, so too must our understanding and regulation of placebo use, ensuring that it serves the best interests of patients while upholding the highest ethical standards.

This contribution focuses on the legal and regulatory frameworks that regulate the use of placebos in medical settings by means of a multifaceted methodological approach. At the heart of our investigation lies an exploration of the legal frameworks that regulate placebo use across the legal systems of France, Germany, Switzerland, United Kingdom, and the United States of America, identifying key statutes, federal acts, and healthcare regulations that directly or indirectly influence the use of placebos in clinical practice. Our exploration covered the legal frameworks in these countries chosen for their legal system diversity, healthcare policy variability, and significant roles in medical research.

## 2 Methods

We examined binding legal (i.e., hard laws) as well as non-binding principles/guidelines/by-laws (i.e., soft laws) (Pronto, 2015) related to placebo usage in France, Germany, United Kingdom, the United States of America and Switzerland, with a focus on Switzerland.

**Figure 1:**
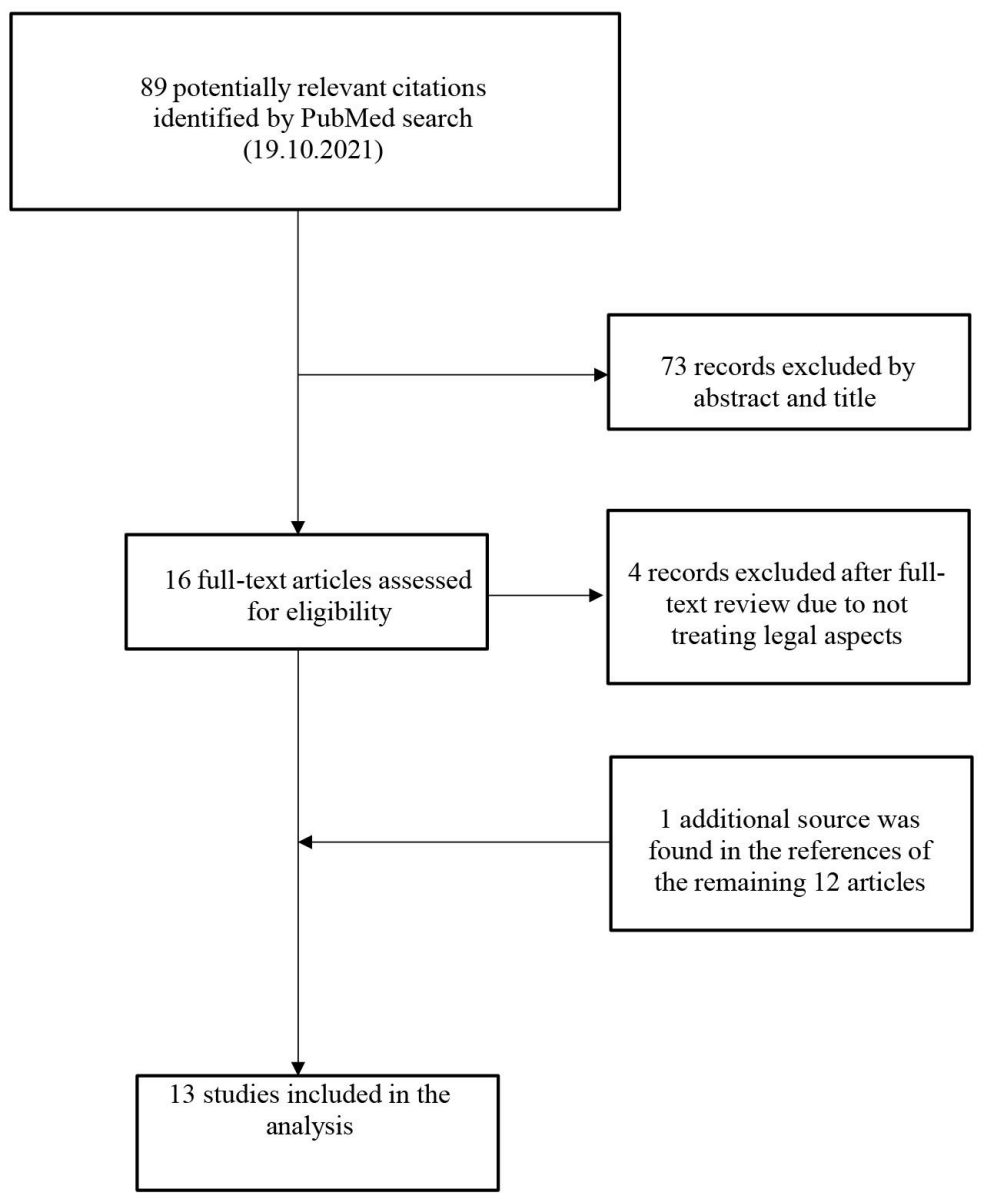
Flowchart of the search process on PubMed for articles on legal aspects with placebo.

**Figure 2:**
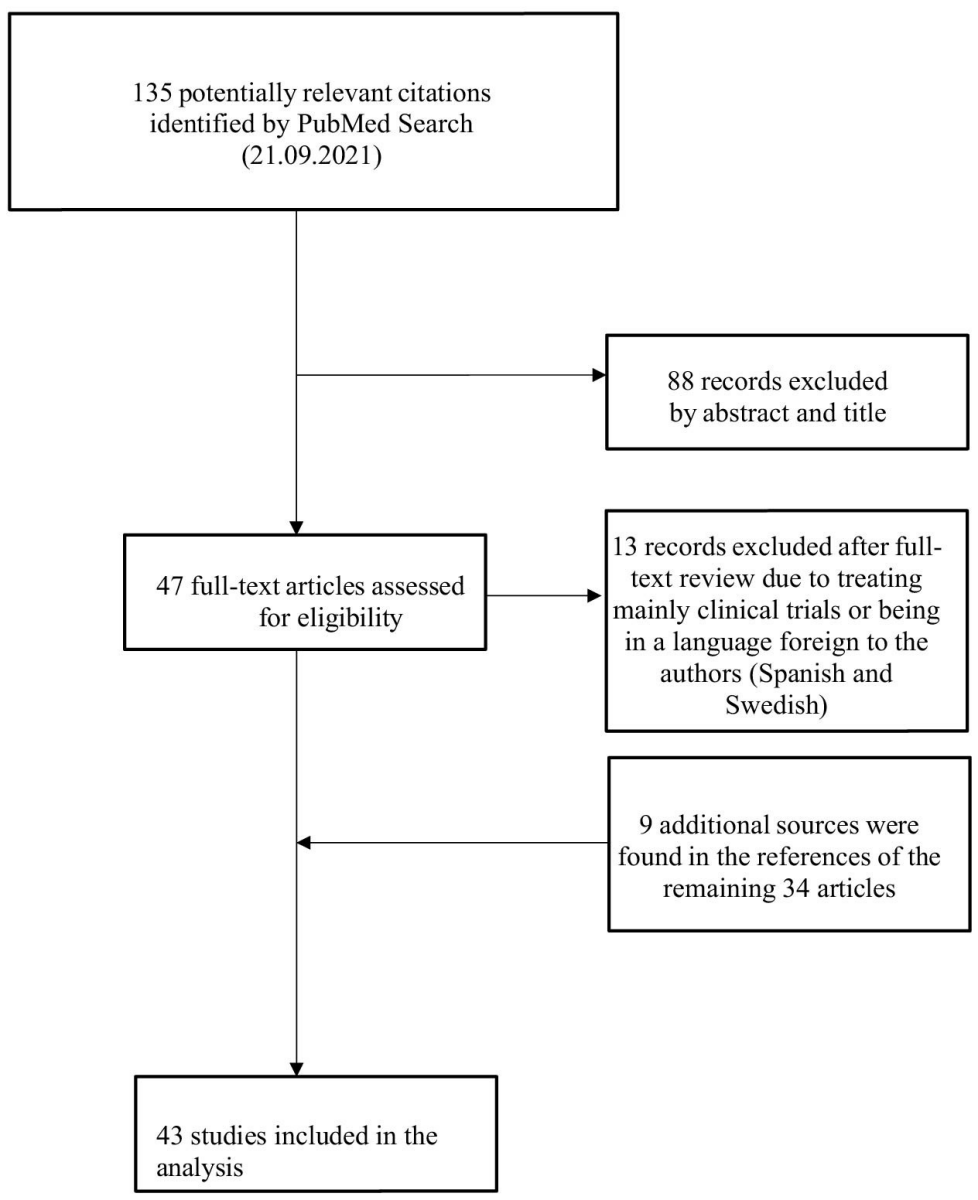
Flowchart of the search process on PubMed for articles on ethical aspects with placebo.

First, hard laws were identified in official legal documents via a thorough review of information from regulatory bodies available online. Second, to understand the current guidelines governing the use of placebos we conducted a literature search focusing on scientific publications related to the legislative aspects of placebo use. To find relevant articles, the search-string (placebo[tiab]) AND [(law[tiab]) OR (legal[tiab])] NOT [(randomized controlled trial) OR (trial)] was used on PubMed. After closer investigation of the 89 articles found, a final selection resulted in 13 relevant titles being included. A flowchart of the search process is presented in Figure 1. Additionally, we analyzed health institution websites for guidelines, codes of conduct, or recommendations on placebo use, focusing on their ethical and moral value in clinical practice. Since laws pertaining to medical care and codes of conduct for healthcare professionals are inherently linked to ethics, a second search on PubMed was conducted to find information about the ethical aspects of placebos in clinical practice. The search string used was [(placebo[tiab]) AND (ethics[tiab])] NOT [(randomized controlled trial) OR (trial)]. One hundred and thirty-five citations were found to match this time. Out of 135 citations identified, 88 were excluded based on abstracts and titles. Of the 47 full-text articles assessed, 13 were excluded for focusing on clinical trials or being in foreign languages. Nine additional sources were identified from the references of the remaining 34 articles, resulting in 43 studies included in the analysis. Figure 2 shows a flowchart of the search procedure.

Hard laws—binding legislative provisions such as duties, rights, and responsibilities—were examined through constitutions, acts, laws, and codes, using keywords placebo and placebos. Codes of conduct, bylaws, and guidelines (soft laws) were also reviewed. Although not legally binding, these soft laws strongly influence jurisprudence, particularly for healthcare professionals. Soft laws include mandatory bylaws, where violations may lead to loss of medical license, and guidelines aimed at improving medical interventions, reflecting ethical and moral expectations. In the focal case of Switzerland, additional investigations were undertaken: email requests were sent to the included health institutions which contained a description of the articles' interest and the inquiry for information about official positions and standards concerning the use of placebo in clinical practice (for more information see Supplementary Table 1S).

**Figure 3:**
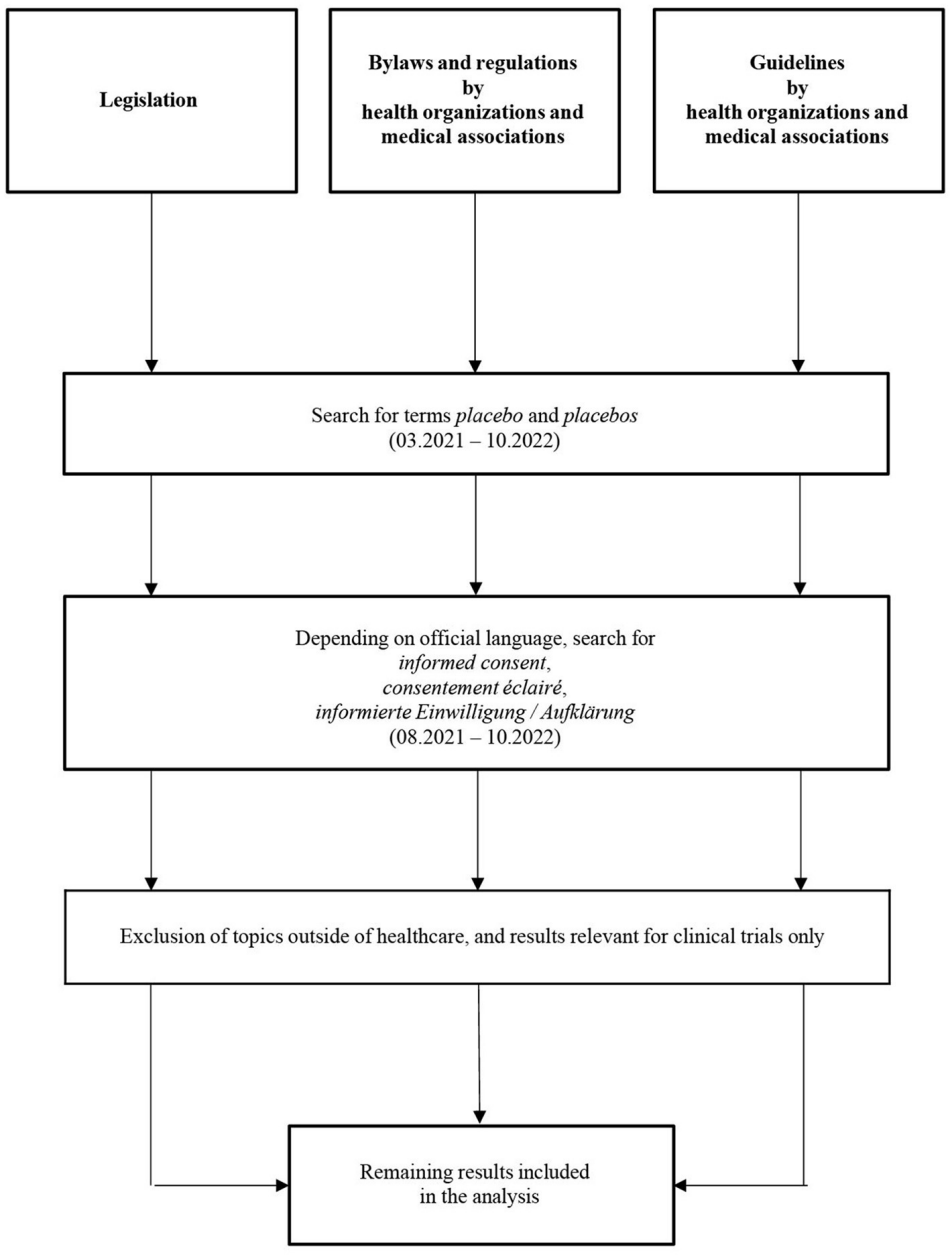
Flowchart of the search strategy for legal regulations, codes of conduct, and guidelines.

Finally, the search process's parameters were expanded to more widely encompass laws pertaining to patients' rights and physicians' obligations to inform patients. For this purpose, the same sources that were used for laws, ethical standards, and guidelines for placebos were examined. However, this time, the search phrases informed consent, consentement éclairé, or informierte Einwilligung/Aufklärung were used, depending on official language and expressing the same idea, respectively. A flow chart of the search strategy is presented in Figure 3.

## 3 Results

The subsequent subsections offer an in-depth look at the legal frameworks established by hard- and soft laws from governments, medical associations, and health organizations. Additionally, this analysis highlights the indirect regulation of placebos through laws and rules that, while not explicitly mentioning placebos, significantly influence their application. An overview table of the regulations and laws on placebos in clinical practice is provided for each country.

### 3.1 Switzerland

The Swiss Academy for Medical Sciences (Schweizerische Akademie der Medizinischen Wissenschaften, SAMW) and the Swiss Medical Association (Verbindung der Schweizer Ärztinnen und Ärzte, FMH) outline the legal framework in Switzerland as hierarchical, with regulations becoming more specific at lower levels. The Federal Constitution of the Swiss Confederation (Bundesverfassung der Schweizerischen Eidgenossenschaft, BV) is at the top, followed by Federal Acts (Bundesgesetz, BG) and the SAMW and FMH's regulations at the base. Regarding the latter, these soft laws significantly influence jurisprudence and legislation (FMH/SAMW, 2020).

In Switzerland, neither the Swiss Academy for Medical Sciences, the Swiss Medical Association or other authorities specifically address the use of placebos in clinical practice, focusing instead on the legal and regulatory adherence to informed consent. Switzerland ratified the Convention on Human Rights and Biomedicine (Übereinkommen über Menschenrechte und Biomedizin) mandating informed consent for healthcare interventions (SR 0.810.2—Kap. II, Art. 5). The Federal Constitution of the Swiss Confederation emphasizes personal liberty, including the right to physical and mental integrity (Constitution, 1999). The Office of Public Health (Bundesamt für Gesundheit, BAG) acknowledges the complexity and variability of informed consent laws, providing further specifications on patient rights in practical situations on their website. This includes the right to information and the requirement for free, informed consent before any treatment or care (BAG, 2015, 2022, September 6). The Swiss Academy for Medical Sciences and the Swiss Medical Association underscore the physicians' duty to inform patients, a responsibility based on numerous legal requirements at both cantonal and federal levels. Any medical treatment without the patient's consent is deemed unlawful and punishable (FMH/SAMW, 2020 p. 36, ch. 3.2; FMH/SAMW, 2020 p. 40, ch. 3.3). Physicians are granted discretion in the amount of information they provide to patients, aiming for a balance in the delivery of necessary information (FMH, 1997) Art. 10).

Additionally, the Swiss Academy for Medical Sciences and the Swiss Medical Association highlight that “Off-label use,” “Unlicensed use,” and “Compassionate use” of medications are not explicitly mentioned in the “Heilmittelgesetz” but are permissible as part of a physician's therapeutic freedom. This includes the administration of pharmaceutical drugs for unapproved indications or without authorization outside of clinical trials (FMH/SAMW, 2020)p. 64, ch. 3.10; (SAMW, 2015). Table 1 shows a summary of the regulations and laws on placebo usage in clinical practice in Switzerland.

**Table 1:** Regulations and laws on placebos in clinical practice in Switzerland.

**Category**	**Source (Original/English)**	**Paragraph**	**Core statement**	**Liability**
International treaty	Übereinkommen über Menschenrechte und Biomedizin [Convention on Human Rights and Biomedicine (also known as the Oviedo Convention)] https://www.fedlex.admin.ch/eli/cc/2008/718/de#chap_I_I	Art. 5	Need for informed consent	Binding
Catalog of fundamental rights	Bundesverfassung der Schweizerischen Eidgenossenschaft Grundrechte, Bürgerrechte und Sozialziele (Federal Constitution of the Swiss Confederation: Fundamental Rights, Civil Rights, and Social Goals) https://www.fedlex.admin.ch/eli/cc/1999/404/de#art_10	Art. 10, Abs. 2	Right to life and personal liberty	Binding
Personality rights	Schweizerisches Zivilgesetzbuch (Swiss Civil Code) https://www.fedlex.admin.ch/eli/cc/24/233_245_233/de#art_28	Art. 28, Abs. 2	Personality infringement hast to be justified by consent	Binding
Guideline	BAGPatientenrechte und Patientenpartizipation (Patient Rights and Patient Participation) https://www.bag.admin.ch/bag/de/home/medizin-und-forschung/patientenrechte.html	1, 2	Need for informed consent	Recommendation
Guideline	SAMW/FMHRechtliche Grundlagen im medizinischen Alltag. Ein Leitfaden für die Praxis (Legal Foundations in Everyday Medical Practice: A Guide for Practitioners) https://doi.org/10.5281/zenodo.3635309	Kap. 3.2; Kap. 3.3	Need for informed consent	Recommendation
Code of Professional Conduct	FMH https://www.fmh.ch/files/pdf7/standesordnung-fmh.pdf	Art. 10	Obligation to inform	Binding for members

Besides Swissmedic, the Swiss national authorization and supervisory authority for drugs and medical products, the web search uncovered regulations on informed consent in clinical practice for all analyzed institutions, detailed in Table 2. Swissmedic lacks documents on informed consent within clinical practice, concentrating instead on clinical research and thus only providing regulations for informed consent in that context. While laws were initially excluded from this study, the informed consent text passages provided by the BAG, which are based on legal provisions, were included. These passages, while not legislative texts themselves, distill the essence of the laws in a manner accessible to those without legal expertise.

**Table 2:** Results of web search for the word “informed consent” in Swiss health authorities and institutions.

**Institution/full name/English translation**	**Source**	**Paragraph**	**Core statement**	**Liability**
BAG/Bundesamt für Gesundheit/Federal Office of Public Health (FOPH)	Ihre Rechte bei einer medizinischen Behandlung. https://www.bag.admin.ch/bag/de/home/medizin-und-forschung/patientenrechte/rechte-arzt-spital.html	1	Right to be informed	Binding
Swissmedic/Schweizerisches Heilmittelinstitut/Swiss Agency for Therapeutic Products	https://www.swissmedic.ch/swissmedic/en/home.html	N/A	N/A	N/A
FMH/Verbindung der Schweizer Ärztinnen und Ärzte/The Swiss Medical Association	FMH Standesordnung https://www.fmh.ch/files/pdf7/standesordnung-fmh.pdf	Art. 10	Duty of disclosure	Binding for members
SAWM/Schweizerische Akademie der Medizinischen Wissenschaften/Swiss Academy of Medical Sciences	Rechtliche Grundlagen im medizinischen Alltag. Ein Leitfaden für die Praxis https://doi.org/10.5281/zenodo.3635309	Kap. 3.2; Kap 3.3	Need for informed consent	Binding for members
FMCH/Verbindung der Schweizer Chirurgen und Chirurginnen/Association of Swiss Surgeons	Richtlinien der FMCH für die Patienten Aufklärung; https://fmch.ch/wp-content/uploads/2020/04/Richtlinien_Aufklaerung_FMCH_DE.pdf	I, II	Need for informed consent	N/A
FMPP/Föderation Medizinischer Psychotherapeutinnen und Psychotherapeuten/Federation of Medical Psychotherapists	Qualität in der ambulanten Behandlung der Kinder- und Erwachsenentherapie; https://www.psychiatrie.ch/fileadmin/user_upload/d_Q-Bericht_ambulanter_Bereich_def_Oktober_2016.pdf; Empfehlung der SGPP und der SGKJPP für die Verwendung der ≪proCOmpliance; Dokumentierte Patientenaufklärung≫; https://www.psychiatrie.ch/fileadmin/user_upload/d_proCompliance_dokumentierte_Patientenaufklaerung.pdf; Erläuterung zur Patientenaufklärung und –dokumentation; https://www.psychiatrie.ch/fileadmin/user_upload/d_Erlaeuterungen_Patientenaufklaerung.pdf	Kap 2.1; 1; 1; 2	Importance of informed Conseco; Need for informed consent; Need for informed consent	Guidance for members recommendation; Binding for members
FSP/Föderation der Schweizer Psychologinnen und Psychologen/Federation of Swiss Psychologists	Berufsordnung; https://www.psychologie.ch/sites/default/files/media-files/2019-07/rz_19fsp_berufsordnung_4sprachig_web.pdf	Art. 11; Art. 30	Need for informed consent	Binding for members
SGP/Schweizerische Gesellschaft für Psychiatrie und Psychotherapie/Swiss Society of Psychiatry and Psychotherapy	Ethische Richtlinien; https://www.swisspsychologicalsociety.ch/fileadmin/user_upload/PDF-Dateien/Ethic_Guidelines/DE/d3-ethische_richlinien18.dt_.pdf	C2	Freedom of information	Binding for members
ASP/Assoziation Schweizer Psychotherapeutinnen und Psychotherapeuten/Association of Swiss Psychotherapists	ASP Standesregeln; https://psychotherapie.ch/wsp/site/assets/files/1041/de_standesregeln-asp-2018.pdf	Kap. 3.1	Duty to inform	Binding for members

Email inquiries regarding placebo usage yielded almost no results: no federal institution or health association provided documents containing placebo regulations in clinical practice. However, the responses did articulate specific stances on placebo use. Key points from these replies are summarized, with an overview in Table 3.

**Table 3:** Public statements of Swiss authorities concerning patient information.

BAG/Bundesamt für Gesundheit/Federal Office of Public Health (FOPH)	1. The right to be informed Patients have the right to be clearly and adequately informed about (…) the planned examinations and treatments, their possible consequences and risks (…). In practice The health professional is obliged to inform patients on his/her own initiative. He/she must provide all the necessary information in a factual and complete manner so that patients can give informed consent to the treatment. (…) Limitation of the right to information The right to be informed can be restricted in two cases - Patients explicitly waive the right to be informed, for example because they do not want to know whether they are suffering from an incurable disease. (…)	1. Das Recht auf Aufklärung Patientinnen und Patienten haben das Recht, klar und angemessen über (…) die geplanten Untersuchungen und Behandlungen, deren allfällige Folgen und Risiken (…) informiert zu werden. In der Praxis Die Gesundheitsfachperson ist verpflichtet, Patientinnen und Patienten von sich aus aufzuklären. Sie muss auf sachliche und vollständige Weise alle nötigen Informationen geben, damit Patientinnen und Patienten in Kenntnis aller Tatsachen der Behandlung zustimmen können. (…) Einschränkung des Rechts auf Aufklärung Das Recht auf Aufklärung kann in zwei Fällen eingeschränkt werden Patientinnen oder Patienten verzichten explizit darauf, aufgeklärt zu werden, zum Beispiel weil sie nicht wissen wollen, ob sie an einer unheilbaren Krankheit leiden. (…)
SGP/Schweizerische Gesellschaft für Psychiatrie und Psychotherapie/Swiss Society of Psychiatry and Psychotherapy	“C2. Psychologists shall take care not to restrict the right of self-determination of fellow human beings. In particular, they shall respect the freedom of information, judgement and decision.”	≪C2. Psychologinnen und Psychologen hüten sich davor, das Selbstbestimmungsrecht von Mitmenschen einzuschränken. Insbesondere achten sie auf die Freiheit der Information, des Urteils und der Entscheidung.≫
ASP/Assoziation Schweizer Psychotherapeutinnen und Psychotherapeuten/Association of Swiss Psychotherapists	“3.1 Duty for patient's information Patients or their legal representative must be orientated specially about the following points: - The type of the method, the setting (…) The patient's orientation about the condition in psychotherapy shall be indicated in a professional, truthful, and appropriate way.”	≪3.1 Informationspflicht gegenüber Patienten/innen Insbesondere sollen PatientInnen bzw. deren gesetzliche/r Vertreter/in über folgende Punkte orientiert werden - die Art der Methode, des Settings (…) Die Orientierung von PatientInnen über die Bedingungen einer Psychotherapie hat sachlich, ehrlich und verhältnismässig zu erfolgen.≫
FSP/Föderation der Schweizer Psychologinnen und Psychologen/Federation of Swiss Psychologists	“Art. 11 Behavior toward clients and patients (…) Members educate their clients and patients or their legal representatives adequately and in comprehensible and professional manner particularly about the nature and extent of intended diagnostic, therapeutic or other procedures and methods. Members shall conduct the information with the necessary care. They shall endeavor to avoid unnecessary stress for clients or patients.”	≪Art. 11 Verhalten gegenüber Klienten/innen und Patienten/innen (…) Mitglieder klären ihre Klientinnen und Klienten oder Patientinnen und Patienten bzw. deren gesetzliche Vertretung in verständlicher und sachlicher Form hinreichend auf, insbesondere über Art und Umfang der beabsichtigten diagnostischen, therapeutischen oder anderen Verfahren oder Methoden. Mitglieder führen das Aufklärungsgespräch mit der nötigen Sorgfalt durch. Sie sind bestrebt, dabei unnötige Belastungen der Klientinnen und Klienten oder Patientinnen und Patienten zu vermeiden.≫
FMH/Verbindung der Schweizer Ärztinnen und Ärzte/The Swiss Medical Association	“Art. 10 Duty of information Physicians shall inform their patients in a comprehensible form about the findings, the intended diagnostic and therapeutic measures, their prospects of success and risks, as well as any treatment alternatives.	≪Art. 10 Aufklärungspflicht Arzt und Ärztin klären ihre Patienten und Patientinnen in verständlicher Form über den Befund, die beabsichtigten diagnostischen und therapeutischen Massnahmen, deren Erfolgsaussichten und Risiken sowie über allfällige Behandlungsalternativen auf. Sie wägen sorgfältig ab, auf welche Art und Weise sie das Aufklärungsgespräch führen und wie viel Informationen sie ihren Patienten und Patientinnen zumuten können.≫

### 3.2 France

In France, there is no specific law directly addressing the administration of placebos in medical practice. However, articles such as Art. L. 1111-4 and Art. L. 1111-2 from the Loi no. 2002-303 du 4 mars 2002 (Republic, 2002) emphasize the necessity of informed consent and transparency regarding the clinical utility of treatments. This legislative context suggests that the deceptive use of placebos may not align with French law (Guimet, 2011).

Despite the lack of legal recommendations in this matter, the use of placebo is included and defined in the French medical curriculum (Guimet, 2011, p. 13). Indeed, the current bylaw for the second round of postgraduate medical studies (Arrêté du 4 mars 1997 relatif à la deuxième partie du deuxième cycle des études médicales, sec. 168) as well as the draft of a reform dated June 2022 (Thérapeutique, 2022) (Programme de connaissances du 2ème cycle, 2022, sec. 323) specifically state that in addition to discussing the use of placebo drugs in clinical research and medical practice, they also explicitly state that the placebo effect and placebo drugs (médicaments placebo) will be examined.

An open-label, pure placebo known as Lobepac—an anagram of placebo—was introduced in France in 2003 with the intention of standardizing the use of placebos for healthcare professionals and increasing patient transparency (Aulas, 2003). It was marketed as a “psycho-active elixir” and came in two flavors: blue for sedatives and red for tonics. Both flavors clearly stated that the contents were inactive. However, it was quickly withdrawn owing to low demand (Aulas, 2003). Table 4 summarizes the regulations and laws on placebos in clinical practice in France.

**Table 4:** Regulations and laws on placebos in clinical practice in France.

**Category**	**Source**	**Paragraph**	**Core statement**	**Liability**
Civil law	Loi n°2002-303 du 4 mars 2002, LOI relative aux droits des malades et à la qualité du système de santé https://www.legifrance.gouv.fr/jorf/id/JORFTEXT000000227015/	Art. L. 1111-4.	Need for informed consent	Binding
Civil law	Loi n°2002-303 du 4 mars 2002, LOI relative aux droits des malades et à la qualité du système de santé https://www.legifrance.gouv.fr/jorf/id/JORFTEXT000000227015/	Art. L. 1111-2.	Transparency on treatment utility	Binding
Decree	Décret n° 2006-1498 du 29 novembre 2006 déterminant les règles selon lesquelles certaines catégories de préparations magistrales et officinales peuvent être exclues du remboursement et modifiant le code de la sécurité sociale (deuxième partie: Décrets en Conseil d'État) – Légifrance https://www.legifrance.gouv.fr/jorf/id/JORFTEXT000000274530	Art. 1	Reimbursement for medications of proven utility only	Binding

### 3.3 Germany

German legislation does not explicitly mention the use of placebos. Instead, it is indirectly governed by laws requiring informed consent for medical interventions, as stipulated in the German Civil Code (Bürgerliches Gesetzbuch, BGB) and its Patient Rights Act (Patientenrechtegesetz). The German Civil Code mandates health practitioners to inform patients about the modalities of any medical intervention and obtain consent prior to proceeding. It details the obligation of disclosure about the intervention's type, scope, implementation, expected outcomes, risks, necessity, and alternatives if applicable (BGB, 2024).

The German Medical Association (Bundesärztekammer, 2021) in the Code of Professional Conduct emphasizes the preservation of patient autonomy, stating that any treatment must respect the patient's right of self-determination.

Administering placebos without disclosure could lead to criminal sanctions, as interpreted under the German Criminal Code (Bundesamt für Justiz, 2024), potentially viewed as a crime against physical integrity and fraud. However, a notable 1988 ruling by the Higher Regional Court of Hamm deemed the use of a placebo lawful under certain conditions: it was based on presumed consent, was not classified as bodily injury, and was not considered fraud (Joerden, 2004). Table 5 summarizes the regulations and laws on placebos in clinical practice in Germany.

**Table 5:** Regulations and laws on placebos in clinical practice in Germany.

**Category**	**Source**	**Paragraph**	**Core statement**	**Liability**
Civil law	German Civil Code (BGB)—Patient Rights Act: Bürgerliches Gesetzbuch (BGB)—Patientenrechtegesetz https://www.gesetze-im-internet.de/bgb/BJNR001950896.html#BJNR001950896BJNG026900377	§ 630d, Abs. 1; § 630e, Abs. 1	Need for informed consent	Binding
Code of Professional Conduct	German Medical Association—Duties Toward Patients: Bundesärztekammer—Pflichten gegenüber Patientinnen und Patienten; https://www.bundesaerztekammer.de/fileadmin/user_upload/_old-files/downloads/pdf-Ordner/Recht/MBO-AE_Beschluesse_124._DAET_2021_engl._Fassung.pdf	§ 7, Abs. 1, S. 9; § 8, S. 10	Obligation of disclosure, informed consent, patient autonomy	Binding for all physicians practicing in Germany
Criminal law	Criminal Code—Offenses Against Physical Integrity: Strafgesetzbuch—Straftaten gegen die körperliche Unversehrtheit https://www.gesetze-im-internet.de/stgb/BJNR001270871.html#BJNR001270871BJNG005403307	§§ 223	Crimes against physical integrity	Binding
Criminal law	Criminal Code—Fraud and Breach of Trust: Strafgesetzbuch—Betrug und Untreue https://www.gesetze-im-internet.de/stgb/BJNR001270871.html#BJNR001270871BJNG005902307	§§ 263	Fraud and embezzlement	Binding

### 3.4 United Kingdom

In the legal framework of the United Kingdom, the administration of placebos in clinical trials is governed by specific legislation, notably the Medicines for Human Use (Clinical Trials) Regulations 2004, as per The National Archives (TNA). Additionally, the Health and Social Care Act mandates explicit patient consent for all medical interventions, emphasizing that care and treatment can only proceed with the agreement of the involved individual (England, 2008) Part 3, Sec. 2, Reg. 11). Contrastingly, guidance on the use of placebos outside clinical trials, particularly for general practitioners, is not explicitly addressed by key medical regulatory bodies such as the General Medical Council (GMC) or the British Medical Association (BMA). However, the legal requirement for patient consent in the UK broadly encompasses the administration of placebos. The GMC, recognizing the importance of consent, provides further instruction to medical professionals. Its publications “Good Medical Practice” (Council, 2013–2024) and “Decision Making and Consent” (Council, 2020) underline the necessity of working in partnership with patients, ensuring they are well-informed about their condition, treatment options, and associated risks or uncertainties before making any healthcare decisions. Furthermore, the GMC advises that while immediate disclosure of all relevant information to patients is preferable, there might be situations where delaying this disclosure is considered appropriate. In such cases, it is recommended that the patient is informed about the impending discussion, with arrangements made for the full disclosure of information at a later, suitable time. Documentation of what information is yet to be shared, the rationale for the delay, and the timeline for future disclosure is also advised (GMC, “Decision making and consent,” Sec. 14, p. 13, 2020).

The GMC clarifies that these guidelines align with the legal standards across all UK nations, supporting doctors to comply with the law (Council, 2013–2024, p. 4). Adherence to these guidelines is rooted in professional judgment and good faith. Nevertheless, only significant, or repeated non-compliance, which endangers patient safety or public confidence in the medical profession, would jeopardize a doctor's registration (Council, 2020, p. 5). This nuanced position suggests a degree of flexibility regarding the use of placebos, provided that the overarching principle of informed consent and patient partnership is maintained. Table 6 summarizes the regulations and laws on placebos in clinical practice in the United Kingdom.

**Table 6:** Regulations and laws on placebos in clinical practice in the UK.

**Category**	**Source**	**Paragraph**	**Core statement**	**Liability**
Primary and secondary legislation	UK Statutory Instruments The Medicines for Human Use, Regulations 2004 (SI 2004/1031) https://www.legislation.gov.uk/uksi/2004/1031/contents?text=placebo#match-1	Part 1, Sec. 2 Part 5, Sec. 35; Sched. 3, Part 2, Sec.; 11(1)	For placebo-controlled trials only	Binding laws and regulations
Primary and secondary legislation	UK Statutory Instruments; The Health and Social Care Act 2008, Regulations 2014 (SI 2014/2936) https://www.legislation.gov.uk/uksi/2014/2936/regulation/11/made	Part 3, Sec. 2, Reg. 11, Par. 1	Need for consent	Binding laws and regulations
Guidance for members	British Medical Association Prescribing in general practice, 04.2018; https://www.bma.org.uk/media/1563/bma-prescribing-in-general-practice-april-https://www.bma.org.uk/media/1563/bma-prescribing-in-general-practice-april-2018.pdf	N/A	N/A	Advice (accessed on 01.10.2022)
Ethical guidance for doctors	General Medical Council; Good medical practice, 03.2013; https://www.gmc-uk.org/-/media/documents/good-medical-practice—english-20200128_pdf-51527435.pdf	49a	Informed consent	Guidance on good practice for members of the GMC
Ethical guidance for doctors	General Medical Council; Decision making and consent, 09.2020; https://www.gmc-uk.org/-/media/documents/gmc-guidance-for-doctors—decision-making-and-consent-english_pdf-84191055.pdf	10, 14	Informed consent, delayed sharing of information	Guidance on good practice for members of the GMC

### 3.5 United States of America

Within the scope of the surveyed countries, only the American Medical Association (AMA) of the United States has established specific guidelines for the use of placebos in clinical practice. These guidelines are articulated in the AMA's Principles of Medical Ethics, incorporated into the Principles of Medical Ethics (AMA, 2016). According to these principles, physicians may utilize placebos in diagnosis or treatment under certain conditions: they must seek the patient's cooperation, explaining the potential benefits of evaluating different medications, including placebos; obtain general consent for placebo use without needing to specify its timing; and refrain from using placebos merely to placate challenging patients. The AMA emphasizes the importance of prioritizing patient welfare over physician convenience and suggests that a placebo-like effect can also be achieved through reassurance and encouragement, thus enhancing the patient-physician relationship (AMA, 2016, Chapter 2, Opinion 2.1.4). On the other hand, the AMA's guidelines stipulate a narrower leeway regarding the disclosure of information about treatment compared to the British General Medical Council. The AMA asserts that withholding information from the patient without their knowledge or consent is ethically unacceptable outside emergency situations where the patient is incapable of making an informed decision. In such cases, information must be disclosed once the emergency is resolved (AMA, 2016, Chapter 2, Opinion 2.1.3).

Although these guidelines serve as ethical guidance rather than establishing clinical or legal standards, non-compliance with the AMA's ethical standards may still jeopardize a practitioner's registration. Legal mandates for informed consent in the U.S. are predominantly governed at the state level, with three prevailing standards: the subjective standard focuses on the individual patient's needs for making informed decisions; the reasonable patient standard considers what an average patient would need to know; and the reasonable physician standard is based on what a typical physician would disclose about a procedure. The reasonable patient standard is widely adopted, as it centers on the information a typical patient would require making an informed decision (Shah et al., 2024). This approach underscores the emphasis on patient autonomy and informed consent within the diverse legal landscape of the U.S. Table 7 summarizes the regulations and laws on placebos in clinical practice in the U.S.

**Table 7:** Regulations and laws on placebos in clinical practice in the USA.

**Category**	**Source**	**Paragraph**	**Core statement**	**Liability**
Ethical guidance for doctors	American Medical Association Principles of Medical Ethics: I, III, V,VIII (2016); https://www.ama-assn.org/system/files/2019-06/code-of-medical-ethics-chapter-2.pdf	Chapter 2, Opinion 2.1.4	Informed consent when using placebos	Guidance for members of the AMA
Ethical guidance for doctors	American Medical Association Principles of Medical Ethics: I,III,V,VIII (2016); https://www.ama-assn.org/system/files/2019-06/code-of-medical-ethics-chapter-2.pdf	Chapter 2, Opinion 2.1.3	Delayed sharing of information in emergency only	Guidance for members of the AMA
Regulation	Code of Federal Regulations; Public Welfare, Protection of Human Subjects (2018) https://www.govinfo.gov/app/details/CFR-2018-title45-vol1/CFR-2018-title45-vol1-sec46-116	Tit. 45, Subt. A, Subch. A, Part 46, Subpart A, § 46.116	Need for informed consent in research only	Binding regulation
Code of Laws	United States Code Veterans' Benefits (2020); https://www.govinfo.gov/app/details/USCODE-2020-title38/USCODE-2020-title38-partV-chap73-subchapIII-sec7331	Tit. 38, Part V, Ch. 73, Sub-Ch. III, §; 7331	Need for informed consent for veterans only	Binding law

## 4 Discussion

The objective of our study was to explore the legal frameworks governing the clinical use of placebos, spotlighting both hard and soft laws in the USA, GB, France, Germany and Switzerland. This initiative aimed at dissecting the legal narratives enveloping placebo use in medical settings, straddling the realms of pure placebos, impure placebos, and notably, OLPs. Our aim was not to unfurl a legal treatise but to shed light on the legal and ethical scaffolding from a clinical vantage point, advocating for a more explicit and informed discussion on placebo use in medical practice.

While all investigated countries emphasize the necessity of informed consent as a cornerstone of medical ethics and practice, the approaches to disclosure regarding placebo use vary significantly. In Switzerland and France, the lack of explicit legislation on placebos leads to a reliance on broader informed consent laws and soft guidelines from medical associations. This absence of specific regulations creates a degree of ambiguity for healthcare providers, potentially leading to inconsistent practices regarding the administration of placebos. In contrast, Germany's legal framework establishes clearer consequences for administering placebos without disclosure, categorizing such actions under the potential for criminal sanctions related to patient autonomy and physical integrity. This more stringent approach places greater emphasis on patient rights, suggesting that German healthcare practitioners may navigate placebo use with heightened caution compared to their counterparts in other countries.

The United Kingdom and the United States offer more nuanced positions on disclosure, allowing for flexibility in certain circumstances. The UK's General Medical Council acknowledges situations where immediate disclosure may not be feasible, while the American Medical Association's ethical guidelines provide a framework that prioritizes patient cooperation but warns against withholding information. This introduces a dynamic where healthcare professionals can exercise professional judgment in specific contexts, although it raises questions about the potential risks of undermining patient trust.

In the labyrinth of legal regulations, the discussion on placebo usage in clinical practice occupies a unique position. It is shrouded in layers of historical practice, ethical debates, and the evolving understanding of the placebo effect itself. Our main findings underscore a conspicuous void in explicit legal documentation and guidelines regarding placebo use. Explicit references to the use of placebos within these frameworks are few and far between, often subsumed under broader ethical considerations such as patient autonomy and the imperative of informed consent. This observation extends to the discussion on OLPs, which, despite a burgeoning interest and promising research findings suggesting their efficacy without deception, remain evidently absent from explicit legal or ethical guidelines.

Our study aims to bridge the gap between clinical practice and legal standards concerning the use of placebos, albeit with certain limitations we openly acknowledge. A notable challenge we faced was the absence of explicit mentions of placebos within legal documents, a detail that underscores the complexity of integrating clinical practices with legal frameworks. As the analysis is explicitly not a legal expertise, our analysis may not encapsulate the full legal breadth or employ precise legal jargon, potentially limiting the depth of our legal interpretation. Indeed, the intricate task of legal translation falls more suitably to those with formal legal expertise, as exemplified in Schöni's comprehensive work on the legal aspects of placebo use in Switzerland (Schöni, 2014). However, our primary aim was not to provide a legalistic portrayal of clinical realities but rather to offer an overview of the legal underpinnings from the perspective of a clinician seeking legal guidance in the use of placebo. Another limitation of this study is that it focuses exclusively on Switzerland as a focal case, without applying the same level of detailed analysis to the additional countries included. This approach was necessitated by resource constraints and the authors' affiliation with Switzerland.

The translation of considerations surrounding the placebo discourse into legal and clinical practice is fraught with challenges and the absence of explicit legal guidelines on placebo use reflects a broader struggle to reconcile the dynamic and often subjective nature of clinical practice with the rigid structures of laws and regulations. This struggle is emblematic of the tension between the art and science of medicine—a tension that is particularly palpable in the discourse on placebos.

Even so, we propose that the deployment of placebos—encompassing all varieties such as pure placebos, so-called impure placebos and open-label placebos, as well as the fundamental components driving placebo effects, namely expectation and the patient-practitioner relationship—warrants explicit consideration within both hard and soft laws. These types of interventions are intricately laden with clinical as well as ethical properties, highlighting the profound impact of placebo mechanisms in medical practices. The deceptive use of placebos represents a significant breach of ethical standards, so egregious that it necessitates incorporation into legal frameworks and bylaws. Likewise, the development of open-label placebo offers a rather counterintuitive clinical possibility, which should both be accompanied by a legal framework to both allow and regulate its application in clinical practice. This move is essential not only for upholding ethical principles in medical interventions but also for fostering a transparent, trust-based patient-care provider dynamic. Such regulatory measures would ensure that the therapeutic potential of placebos is harnessed in a manner that respects patient autonomy and informed consent, marking a crucial step toward ethical and legal clarity in medical practices.

## Data Availability

The original contributions presented in the study are included in the article/Supplementary material, further inquiries can be directed to the corresponding author.
