# Supplementary material for: Noble Humbug? Hard and soft laws on clinical placebo use
Source: Front Psychol. 2025 Mar 12;16:1520664. doi: 10.3389/fpsyg.2025.1520664 (PMC11938247; doi:10.3389/fpsyg.2025.1520664)
Supplement: Supplementary file 1 [file Table_1.docx]

Table 1S: Results of e-mail request for regulation concerning placebo

| **Institution** | **Division and Email** | **Document** | **Core statement** | **Liability** | **Date of reply** |
| --- | --- | --- | --- | --- | --- |
| BAG | Therapeutic Products Law Section [hmr@bag.admin.ch](mailto:hmr@bag.admin.ch) | N/A | Placebos are not included in the Therapeutic Products Act and not subject to authorization | N/A | September 15, 2022 |
| Swissmedic | General information / communication [anfragen@swissmedic.ch](mailto:anfragen@swissmedic.ch) | N/A | Placebo itself is not considered for the categorization of a study | N/A | September 7, 2022 |
| FMH | Legal division [lex@fmh.ch](mailto:lex@fmh.ch) | N/A | N/A | N/A | August 29, 2022 |
| FMPP / SGPP | Board of directors [sgpp@psychiatrie.ch](mailto:sgpp@psychiatrie.ch) | N/A | Redirected to the SAMW | N/A | August 25, 2022 |
| SAWM | Resort Ethic [mail@sawm.ch](mailto:mail@sawm.ch) | N/A | Ethical placebo administrations in clinical practice are conceivable | N/A | August 24, 2022 |
| FSP | Legal Division, [recht@fsp.psychologie.ch](mailto:recht@fsp.psychologie.ch) | N/A | no sufficient expertise on topic, redirected to SVKP | N/A | September 3, 2022 |
| SGP | Board of directors sekretariat@ssp-sgp.ch | N/A | Clearly rejects the use of deceptive placebos in clinical practice | Members | September 14, 2022 |
| ASP | asp@psychotherapie.ch | N/A | Psychologists are only allowed to administer a placebo | N/A | September 7, 2022 |
| SVKP | Direction sekretariat@svkp-aspc.ch | N/A | No guidelines available | N/A | September 9, 2022 |

### 
